# 

**Published:** 1818-07-01

**Authors:** 


### THE
### Medico-Chirurgical Journal;

OR,

### Quarterly Register

OF

### MEDICAL AND SURGICAL SCIENCE:

INCLUDING

### MEMOIRS OF NAVAL AND MILITARY MEDICINE:

CONTAINING,

I. Practical Essays and Communications.
II. BIBLIOLOGY; or, Analytical Reviews of all important Medical Publications.
III. Foreign Medical Literature.
IV. The PORT-FOLIO; or, Medical and Surgical Miscellany.

---

CONDUCTED BY

JAMES JOHNSON, M. D.

Surgeon to His Royal Highness the Duke of Clarence; Author of "The Influence of Tropical Climates on European Constitutions;" and of "The Influence of the Atmosphere on the Health and Functions of the Human Frame," &c.

SURGEON GEN'LS OFFICE LIBRARY. 63389

---

VOL. I.

Quarterly Series,

FROM JULY 1818, TO JULY 1819.

---

Sæpe Stylum vertas iterum quæ digna legi sint

Scripturus; neque te ut miretur turba labores ——— Hor.

---

S.G.O. LIBRARY

*LONDON.*

PRINTED FOR THE EDITOR,

BY WILLIAM THORNE, RED LION COURT, FLEET STREET.

Sold by BURGESS and HILL, Windmill Street, Haymarket, (to whom Communcations, Post paid, addressed to the Editor, are requested to be sent); BALDWIN, CRADOCK, and JOY, Pater-Noster Row; HIGHLEY and SON, and T. & G. UNDERWOOD, Fleet Street; J. CALLOW, Crown Court, Princes Street, Soho; COX and SON, Borough; ANDERSON and CHASE, West Smithfield; ADAM BLACK, Edinburgh; HODGES and M'ARTHUR, Dublin; and may be had also by applying to the Clerks of the Roads, at the General Post Office.

### CORRESPONDENCE.

Dr. Porter's promised favour on Hydrocephalus will be thankfully received for the ensuing Number. Dr. Robertson's Case of Enteritis with Dissection, and Humanus "On the Language of Complaint in Pain," are deferred unavoidably; as are "Semeiological Observations," "The Dangers of Dissection," "The Morbid Anatomy of Hæmorrhoids," the long and important Paper on "Gastric Scirrhus." &c. &c.

Mr. Newnham's work on Inversio Uteri will be reviewed in our next; as will Dr. Burrows's work on the Bills of Mortality, &c. &c.

---

*** The Editors pledge themselves that *every Work* which is sent to the Printer's Office, during the first six weeks of each quarter, will be noticed in *that* quarter. If they come later, they will probably be deferred till the succeeding publication.

Dr. James Johnson, the Managing Editor, having fixed his Residence in the Metropolis, will spare no pains to give this Journal every possible development, and hopes, should the medical public encourage his exertions, to remove the complaint, that no good *Quarterly* Medical Journal exists on this side of the Tweed.

No. 1, *Albany, Piccadilly,* 1818.

☞ *Our next Number will be published on the first of October.*

*The following Gentlemen have kindly transmitted their Names to* Dr. JOHNSON *as* additional *Subscribers to the Quarterly Form of the Medico-Chirurgical Journal.*

Adams, Sir W. Albemarle Street
Anderson, Dr. R. N.

Burnett, Dr. Chichester
Best, Mr. Tavistock Street
Bampfield, Mr. Bedford Street
Baynton, Mr. Bristol
Barry, Dr. Clifton
Burnaud, Mr. Chichester
Bishop, Mr. Newman Street
Bartlett, Mr. Ipswich
Bell, Mr. Newry
Bennet, Mr. Shifnal, Salop
Byass, Mr. Arundel

Cooper, Mr. Portsea
Cuthbert, Mr. Suffolk
Candler, Mr. Grundesburgh
Crawford, Mr. Southampton
Cunningham, Mr. H. M. S. Rochefort

Dickson, Dr. Clifton
Denmark, Dr. Caen
Dewar, Dr. Edinburgh
Davie, Mr. Ipswich
Davidson, Mr. Edinburgh

Ford, Mr. Southsea
Felix, Dr. Bristol
Fletcher, Mr. Arundel

Grant, Dr. Picardy Place, Edinburgh
Grelke, Mr. H. M. S. Queen Charlotte
Gunning, Deputy-Inspector, Portsmouth

Hall, Mr. Cape of Good Hope
Howell, Dr. Clifton
Hannay, Mr. Trincomalee, Ceylon
Harding, Mr. Kentish Town
Hunt, Mr. Bombay
Highley, Mr. Surgeon, Elland
Hennen, Deputy-Inspector, Edinburgh
Hogg, Mr. Littlehampton
Hall, Dr. John, Berwick
Hutchison, Mr. A. C. Spring Gardens

Johnston, Kennedy, Dowgate Hill
Jubb, Mr. Halifax

Keddell, Mr. Portsea

Lempriere, Dr. Isle of Wight
Lynn, Mr. Woodbridge
Little, Dr. Tandragee
Lane, Mr. Arundel
Leslie, Mr. H. M. S. Prince
Lyne, Mr. Emsworth

M'Donnel, Dr. Belfast
Medical Society, Chichester
————, Halifax, Yorkshire
Moulson, Dr. Halifax, Yorkshire
Morrison, Dr. Newry
Muttlebury, Dr. Bath
Meek, Mr. Edinburgh
Medical Book Club, Edinburgh
Milne, Dr. Edinburgh
M'Carrogher, Dr. Armagh
Mitchell, Mr. Keighley
M'Arthur, Dr. Deal
Medical Society, Deal and Sandwich
————, Portsmouth
Mardel, Mr. Portsmouth

Nagle, Dr. H. M. S. Vengeur
Nowood, Mr. Dover

Osborne, Mr. H. M. S. Grasshopper

Outhwaite, Mr. Bradford
O'Meara, Mr. St. Helena

Porter, Mr. Portsea
Percival, Dr. G. Bath
Porter, Dr. Bristol
Pener, Mr. West Indies
Pink, Mr. Portsea
Pesket, Mr. East Aspling

Quarrier, Dr. R. M. A.

Ross, Mr. Sterling
Rogers, Mr. H. M. S. Minden
Robertson, Dr. Northampton
Robertson, Dr. Archibald, Edinburgh
Risk, Dr. Plymouth Dock

Smith, Mr. Malta
Sharpe, Mr. Busshire, Persia
Stewart, Mr. H. M. S. Bulwark
Sims, Mr. Southsea
Sutcliffe, Mr. Rochedale
Sanden, Dr. Chichester
Stebbing, Mr. Ipswich
Scudamore, Dr. Wimpole Street
Sanders, Dr. Edinburgh
Scott, Mr. Edinburgh
Steel, Dr. Walter, Edinburgh
Stewart, Mr. and Co. Edinburgh
Shaw, Mr. Halifax
Seeds, Dr. Portsea
Stewart, Mr. New Forest
Skair, Mr. Castle Street, Leicester Square
Stewart, Mr. H. M. S. Carron
Shepherd, Mr. Witney, Oxford
Shoveller, Mr. West Indies
Simpson, Mr. James, Edinburgh

Thomson, Dr. Belfast
Travers, Mr. East Bagholt
Thomas, Mr. H. Leigh, Leicester Place, Leicester Square

Weir, Dr. Victualling Board
Williams, Dr. W. H. Ipswich
Wardrope, Mr. Arundel
Williams, Mr. G. Portsea
Webster, Mr. 51st. Regt. L. I.
Warden, Mr. Appley, Isle of Wight
Wills, Mr. Ratcliffe Highway
Walker, Dr. John, Walbrook,

W. Thorne, Printer,
Red Lion Court, Fleet Street.

